# Study of Podoplanin-Deficient Mouse Bone with Mechanical Stress

**DOI:** 10.3390/dj13020061

**Published:** 2025-01-29

**Authors:** Takenori Kanai, Kyoko Osawa, Koichiro Kajiwara, Yoshiaki Sato, Yoshihiko Sawa

**Affiliations:** 1 Department of Orthodontics, Faculty of Dental Medicine and Graduate School of Dental Medicine, Hokkaido University, Kita 13 Nishi 7, Kita-ku, Sapporo 060-8586, Japan; takenori@den.hokudai.ac.jp (T.K.); k.osawa@den.hokudai.ac.jp (K.O.); yoshi-ma@den.hokudai.ac.jp (Y.S.); 2 Department of Oral Growth & Development, Hokkaido University, Kita 13 Nishi 7, Kita-ku, Sapporo 060-8586, Japan; kajiwak@fdcnet.ac.jp; 3 Department of Oral Function & Anatomy, Okayama University Graduate School of Medicine, Dentistry and Pharmaceutical Sciences, Okayama 700-0914, Japan

**Keywords:** podoplanin, cKO, osteocyte, bone, remodeling

## Abstract

**Objective:** We investigated morphological differences in osteocyte processes between aged mice and our original podoplanin-conditional knockout (cKO) mice in which the floxed exon 3 of podoplanin was deleted by Dmp-1-driven Cre (Dmp1-Cre;PdpnΔ/Δ). **Methods:** SEM observation on osteocyte cell process, histochemistry for bone remodeling with mechanostress, and RT-PCR for RANKL and M-CSF in podoplanin cKO mouse bone with mechanostress was investigated. **Results:** SEM observations showed fewer and thinner osteocyte processes in femurs from 23-week-old Dmp1-Cre;PdpnΔ/Δ mice than from 23-week-old wild-type mice, while the numbers of osteocyte processes in femurs and calvarias were similar in 23-week-old Dmp1-Cre;PdpnΔ/Δ mice and 48-week-old wild-type mice. Furthermore, cell process numbers in femurs and calvarias were significantly smaller in 23-week-old Dmp1-Cre;PdpnΔ/Δ mice than in 48-week-old wild-type mice. In the test for differences in alveolar bone resorption under mechanical stress between Dmp1-Cre;PdpnΔ/Δ and wild-type mice, the area of TRAP-positive resorption pits was larger in wild-type mice than in Dmp1-Cre;PdpnΔ/Δ mice. In a quantitative tissue PCR analysis, the mRNA expression levels of RANKL and M-CSF in alveolar bone under mechanical stress were significantly lower in Dmp1-Cre;PdpnΔ/Δ mice than in wild-type mice. These results suggest that a reduction in cell process formation in osteocytes with podoplanin cKO affected the absorption of alveolar bone under mechanical stress in Dmp1-Cre;PdpnΔ/Δ mice. **Conclusions:** In podoplanin-deficient bone, the deformation of osteocyte processes by mechanical stimuli is not recognized as a stress due to the lower number of cell processes with podoplanin deficiency; therefore, the production of osteoclast migration/differentiation factors by activated osteocytes is not fully induced and macrophage migration to alveolar bone with mechanical stress appeared to be suppressed. These results indicate that podoplanin-dependent osteocyte process formation indirectly plays a key role in sensing mechanical stress in bone.

## 1. Introduction

Podoplanin is a mucin-like platelet aggregation protein with a high content of sialic acid and binds C-type lectin-like receptor-2 (CLEC-2) [1,2]. Since podoplanin is an essential molecule for the differentiation of the podoplanin-positive type I alveolar epithelium, podoplanin-deficient mice have impaired alveolar sac development and die at birth [3,4]. Many studies reported the expression of podoplanin in different organs, such as the kidneys, lungs, bone, and lymphatic vessels, and in cancer [1,2]. We have reported the production of podoplanin in teeth, salivary glands, nervous tissue, and placenta [4]. Podoplanin has a high sialic acid content and strongly binds to positively charged proteins. It is considered to contribute to the metastasis of tumor cells. OTS-8 is the earliest reported gene for podoplanin DNA [5], which was produced in osteoblast-like MC3T3-E1 cells activated with the carcinogenic 12-O-tetradecanoylphorbol-13-acetate. The gp38 is the antigen of mouse thymic epithelial cells for a very famous commercially available hamster monoclonal antibody for mouse podoplanin, which is produced from the clone 8.1.1 [6]. Podoplanin has also been established as an osteocyte marker expressed on the plasma membrane of cell processes in the early stages of differentiation and increases in murine osteoblast-like MC3T3-E1 cells cultured in mineralization medium [7,8,9,10,11]. E11 is a well-known alias of podoplanin that was identified as a target of a monoclonal antibody in the rat osteoblast-like osteosarcoma cell line ROS17/2.8 [9]. Mature osteoblasts and osteocytes express podoplanin in their dendrites, and its level was shown to increase in MC3T3-E1 cells and human osteoblasts cultured in mineralization medium [7,8,9,10,11]. Furthermore, pre-osteocytes were found to have higher expression levels of podoplanin in dendritic processes during differentiation into osteocytes than other osteocyte differentiation markers, such as dentin matrix protein 1 and sclerostin [12]. Cultured murine osteocytes from long bones and calvarial bone express podoplanin more highly than MC3T3-E1 cells [7,8,9,10,11,12]. Since pre-osteocytes highly express podoplanin during differentiation into osteocytes, podoplanin may play a role in metabolic homeostasis in osteocytes and contribute to the formation of cell network [13,14,15,16,17,18].

We recently reported that developmental anomalies in alveolar bone and teeth did not occur in podoplanin gene (*Pdpn*)*^Δ/Δ^*-conditional knockout (cKO) mice generated by mating *Wnt1-Cre* transgenic mice with *Wnt1* promoter-driven Cre recombinase and *Pdpn*^fl/fl^ mice carrying homozygous podoplanin-floxed alleles [4]. In *Wnt1-Cre;Pdpn^Δ/Δ^* mice, teeth and alveolar bone grew without developmental anomalies, indicating that podoplanin is not essential for bone development. Previous studies reported the involvement of podoplanin expression in compressive and shear forces in osteoblasts [19,20]. Podoplanin has been used as an important osteocyte marker and is considered to play important roles in mineralization and mechanotransduction [21]. We demonstrated that osteoblasts under mechanical stress increased the production of podoplanin and extended cell processes, and also that mineralization and cell process elongation in osteoblasts were inhibited by CLEC-2 and antibodies specific for podoplanin [22,23]. Therefore, podoplanin in the osteocyte process may be important for sensing mechanical stress in bone. We recently generated podoplanin-cKO mice in which the floxed exon 3 of podoplanin was deleted by dentin matrix acidic phosphoprotein 1 (*Dmp-1)*-driven Cre (*Dmp1-Cre;Pdpn^Δ/Δ^*) [4]. No morphological differences in bone matrix formation or osteocyte distribution were observed between *Dmp1-Cre;Pdpn^Δ/Δ^* and wild-type mice, whereas the formation of osteocyte processes was sparser and networks with neighboring cells were fewer in *Dmp1-Cre;Pdpn^Δ/Δ^* mice than in wild-type mice, suggesting that podoplanin cKO in osteocytes inhibited osteocyte network formation with cell process elongation [23]. Therefore, the present study investigated morphological differences in osteocytes between aged and *Dmp1-Cre;Pdpn^Δ/Δ^* mice and also examined the effects of mechanical stress induced by orthodontic force on bone in *Dmp1-Cre;Pdpn^Δ/Δ^* mice.

## 2. Materials and Methods

### 2.1. Experimental Ethics

In the present study, we used animals to investigate morphological differences in osteocytes between aged and podoplanin-cKO mice and to examine the effects of mechanical stress induced by orthodontic force on bone in podoplanin-cKO mice. This study was performed following the ARRIVE guidelines for animal use to research mechanostress-induced morphological events in bone in mice in which podoplanin alleles were inactivated in osteocytes and odontoblasts. We followed all applicable international, national, and/or institutional guidelines for animal care, as described elsewhere [23]. All experimental and breeding procedures were in accordance with the ethical standards of the institutional and/or national research committees and with the 1964 Helsinki Declaration and its later amendments or comparable ethical standards. The experimental procedures for animal use in this study were approved by the Animal Experiment Committee of Fukuoka Dental College (No. 19010, approval date: 18 May 2020) and were performed in accordance with relevant guidelines and regulations. We performed all procedures for experiments and animal breeding in the Fukuoka Dental College Animal Center in accordance with the conditions and procedures described elsewhere [23]. A daily assessment for humane endpoints was performed and mice reaching humane endpoints were euthanized by induction anesthesia with intraperitoneal injections of sodium pentobarbital and cervical dislocation. In the present study, there were no exclusions of mice and all employed mouse data were used as experimental and control data.

### 2.2. Generation of Podoplanin-cKO Mice 

The methods for generating embryonic stem (ES) cells and cKO mice carrying the homozygous floxed podoplanin allele (*Pdpn^fl/fl^*) were previously described [4,23]. Briefly, we used the *Pdpn* targeting vector HTGR03003_Z_2_G05 (EUCOMM) carrying the C57BL/6N-A^tm1Brd^ genetic background to generate KO, *Pdpn*^tm1a(EUCOMM)Wtsi^. First, a conventional knock-in-first allele was made by using the allele targeted with the gene trap cassette (*Pdpn^gt^*, TransGenic Inc. Fukuoka, Japan) to disrupt targeted *Pdpn* splicing in C57BL/6N ES cells. The *Pdpn^gt^* was knocked-in the *Pdpn* locus (GeneID: 14726, chromosome 4) by homologous recombination. We used the loxP-sandwiched *Pdpn* gene ENSMUSE00000180432 in the Ensemble database and exon 3 in NM_010329 in the NCBI database. We made knock-in-first allele (*Pdpn*^KO1st^) of the *Pdpn* gene from chimeric mice with *Pdpn*-targeted ES cells carrying the C57BL/6NCrj genetic background. The targeting cassette driven by the *Pdpn* promoter region was flanked by FRT sites targeted with the recognition by flippase (Flp) in the targeted gene and we generated a *Pdpn* cKO allele including loxP sites flanking exon 3 (*Pdpn^fl^*, floxed exon 3) by removal of the targeting cassette by Flp. Since all *Pdpn* transcription variants had exon 3, the deletion of exon 3 was performed by utilizing a frameshift mutation leading to a premature stop codon. The *Pdpn* exon 3 deletion formed a stop codon near the 5′ end of exon 4 or 5, depending on splicing variants, and prematurely caused the disruption of the translation of *Pdpn* (*Pdpn^Δ^*). 

In order to leave *Pdpn^fl^*, mice carrying the heterozygous *Pdpn^gt^* allele were mated with mice with Flp, *ACTB:FLPe* (B6;SJL-Tg(ACTFLPe)9205Dym/J, JAX 003800). The C57BL/6.FVB-Tg (*Dmp1*-cre) 1Jqfe/BwdJ mouse line which has the *Dmp1* promoter-driven heterozygous tissue-specific Cre recombinase gene was mated with mice coincidently expressing homozygous *Pdpn^fl^* alleles (*Pdpn^fl/fl^*) and used as mice with *Pdpn*-cKO in *Dmp1*-expressing osteocytes and odontoblasts (*Dmp1*-*Cre*;*Pdpn^Δ^*).

### 2.3. Breeding and Animal Experiments

We used three types of 4-week-old mice for mating (2 pairs in each): C57BL/6N wild-type mice having wild type podoplanin alleles (Kyudo, Fukuoka, Japan), C57BL/6.FVB-Tg (*Dmp1*-Cre) 1Jqfe/BwdJ (*Dmp1*-Cre)(Jax Strain #023047) (The Jackson Laboratory Japan, Inc, Yokohama, Japan), and C57BL/6N with floxed *Pdpn* exon 3 alleles (*Pdpn^fl/fl^*). We finally acquired *Pdpn^fl/fl^* × *Dmp1-Cre* mice with *Pdpn^Δ/Δ^* alleles in *Dmp1*-expressing cells (*Pdpn^Δ/Δ^*). The genotyping method is described elsewhere [4,23]. Briefly, genomic DNA from the tail was isolated with a QIAamp DNA Blood and Tissue Kit (Qiagen, Hilden, Germany). All procedures were conducted according to protocols provided by the manufacturers. PCR was performed for genomic DNA by 30 cycles of amplification using the Ex Taq hot start version (Takara Bio Inc., Otsu, Japan) with 50 pM of primer sets (Sigma-Aldrich Corp., Tokyo, Japan) and products were separated on 2% agarose gel (NuSieve; FMC, Rockland, ME, USA) and visualized using Syber Green (Takara, Shiga, Japan).

Breeding and animal experiments were conducted in the Fukuoka Dental College Animal Center in accordance with guidelines and regulations for the relevant experimental procedures approved by the Animal Experiment Committee of Fukuoka Dental College (No. 19010), as described above [4,23]. Mice were maintained with normal feeding under conventional conditions in the animal center room with a 100% controlled atmosphere, which had passed an examination for bacteria in the Fukuoka Dental College Animal Center. Mice were housed 2 per cage with a 12 h day/night cycle with lights on from 7:00 p.m. with a temperature of 22 °C and 55% humidity. Humane endpoints were assessed daily for the health status and mice with the inability to take water and food were immediately euthanized by induction anesthesia. All specimens were collected from mice euthanized by induction anesthesia (1 L/min of 2% isoflurane mixed with 30% oxygen and 70% nitrous oxide with an anesthetic apparatus) followed by cervical dislocation and intraperitoneal injections with sodium pentobarbital (150 mg/kg, Sumitomo Dainippon Pharma Co., Ltd., Osaka, Japan) at the end of the designated period of the experiments. In summary, 6 mice in each group of wild-type and *Dmp1-Cre;Pdpn^Δ/Δ^* mice were used to investigate the bone characteristics in 23-week and 48-week-old mice. Furthermore, 6 mice in each group of wild-type and *Dmp1-Cre;Pdpn^Δ/Δ^* mice were investigated for alveolar bone characteristics with stress in 23-week-old mice. Alveolar bone around maxillary incisors was loaded with a compressive orthodontic force by inserting a 1 mm-thick plastic plate between the interdental space of the upper incisors, fixing by adhesive resin for one week, and collecting bone after euthanasia. In each group of wild-type and *Dmp1-Cre;Pdpn^Δ/Δ^* mice, two 1-week-old mice were used to investigate podoplanin expression in teeth and alveolar bone.

### 2.4. Histochemistry

The procedures employed are described elsewhere [4,23]. Morphological changes in bone resorption were investigated in serial sections of the upper incisors from 4-week-old male *Dmp1-Cre;Pdpn^Δ/Δ^* mice using hematoxylin and eosin (HE), Villanueva Osteochrome Bone Stain (Polysciences, Inc., Warrington, PA, USA), and tartrate-resistant acid phosphatase (TRAP) staining with a TRAP Stain Kit (Wako Pure Chemical Industries, Ltd., Osaka, Japan) according to the manufacturers’ instructions. In tissue staining, Kawamoto’s film method with a tungsten carbide blade was used for sectioning to provide intact hard and soft tissues for observations without decalcification [4,23]. After tissues were embedded in super cryoembedding medium (Leica Microsystems Japan, Tokyo, Japan) and rapidly frozen using liquid N_2_, coronal undecalcified frozen sections (10 μm) of tissue, including the upper incisor region, were cut on a cryostat (Leica Microsystems, Wetzlar, Germany) with a tungsten carbide blade. Sections were fixed in 100% ethanol at RT for 30 s and subsequently immersed in 100% methanol at −20 °C for 30 s, treated with 0.1% goat serum at 20 °C for 30 min, and then treated with PBS containing 0.1% goat serum and hamster monoclonal anti-mouse podoplanin from clone 8.1.1 (1 μg/mL, AngioBio Co., Ltd., Del Mar, CA, USA) at 4 °C for 8 h. After the treatment with primary antibodies, sections/cells were washed three times in PBS for 10 min and immunostained at 20 °C for 0.5 h with 0.1 μg/mL of the secondary antibody, Alexa Fluor 488-conjugated goat anti-hamster IgG (Probes Invitrogen Com., Eugene, OR, USA). Immunostained sections were mounted in 50% polyvinylpyrrolidone solution and examined by fluorescence microscopy (BZ-9000, Keyence Corp., Osaka, Japan), confocal laser-scanning microscopy (LSM710, Carl Zeiss, Jena, Germany), or fluorescence microscopy with the Plan Apo lens (Eclipse Ci & DS-Qi2, Nikon, Tokyo, Japan). Bone resorption levels were measured by the total TRAP-stained area, which was assessed in all field-of-view images of 10 sections/mouse (6 mice) by Image J (version 1.54i, National Institutes of Health, Bethesda, MD, USA).

### 2.5. Reverse Transcription (RT)-PCR and Real-Time PCR

The procedures employed are described elsewhere [4,23]. Real-time PCR was performed to quantify the mRNA amounts of RANKL (NM_011613, forward: tgaagacacactacctgactcctg; reverse: gtgactttatgggaacccgatgg, 225 bp) and M-CSF (NM_001113529, forward: gactatgaggagcagaacaaggc; reverse: ctcggctagagcacttagcaaag, 169 bp) in bone tissue using primer sets with specificities that had been confirmed by the manufacturer (Sigma-Aldrich, Tokyo, Japan). Bone tissue squares (side lengths of 5 mm) were collected immediately after excision and ground into a paste with a scalpel on glass plates on ice and dissolved in the RLT buffer of an RNeasy kit (Qiagen, Inc., Tokyo, Japan). Total RNA extraction from tissue was performed with a QIAshredder column and an RNeasy kit (Qiagen, Hong Kong, China). The cDNA samples were analyzed by RT-PCR with 50 pM of primer sets to quantify mRNA amounts. cDNA (1 μL) was amplified in a 25-μL volume of PowerSYBR Green PCR Master Mix (Applied Biosystems, Foster city, CA, USA) in a Stratagene Mx3000P real-time PCR system (Agilent Technologies, Inc., Santa Clara, CA, USA) and fluorescence was monitored in each cycle. Cycle parameters were 95 °C for 15 min to activate Taq followed by 40 cycles at 95 °C for 15s, 58 °C for 1 min, and 72 °C for 1 min. Two standard curves for the real-time analysis of amplicons for β-actin and RANKL/M-CSF genes in three serial 4-fold dilutions of cDNA were quantified to measure RANKL/M-CSF cDNA levels with β-actin/target standard gene curves by allowing Mx3000P software to accurately evaluate each cDNA unit. RANKL and M-CSF cDNA levels in each sample were normalized to β-actin cDNA and expressed in arbitrary units.

### 2.6. Scanning Electron Microscopy (SEM)

The procedures used are described elsewhere [23]. Bone specimens were collected from wild-type and *Pdpn^Δ^/^Δ^* mice and fixed in PBS containing 4% paraformaldehyde. Specimens were demineralized in G-Chelate/Quick at 4 °C for 7 days (GenoStaff Inc., Tokyo, Japan) and neutralized in G-Chelate/NT (GenoStaff) at 4 °C for 12 h. After treatment with 30% potassium hydroxide at 60 °C for 7 min, specimens were dehydrated in ethanol (70, 80, 90 and 100%) at RT for 30 min each and critical point drying was performed by Hitachi HCP-1 at 37 °C in 80 atm for 1 h. Finally, specimens were adhered to sample stages by carbon double-sided tapes, coated with platinum-palladium using a Hitachi E-1030 for 120s, and observed by SEM (Hitachi S4800) with an electron gun acceleration voltage of 5.0 kV at a magnification of ×5.00 k. The osteocyte dendritic network was measured at five different points (0.36 × 0.36 mm^2^) in section images using Image J (National Institutes of Health, Bethesda, MD, USA) according to the procedure described elsewhere [4,23]. Briefly, the margin of the osteocyte in the SEM image was traced on graph paper and the highest, lowest, leftmost, and rightmost points of the cell margin were selected. A rectangle was drawn that touched the cell at the four selected points and the center of the rectangle was determined as the center of the cell. Perpendicular lines were drawn from the center to each side of the rectangle and a square with each side of 3 μm was drawn 3 μm away from the point where the perpendicular lines intersected with the cell margin. The cell process number/μm^2^ within the 3 μm square was measured and the mean of the numbers in four squares was quantitatively determined as the proper number of osteocyte process in the SEM image.

### 2.7. Statistical Analysis

We repeated all experiments ten times and expressed data as the mean + SD. We statistically analyzed data by a one-way ANOVA and the unpaired two-tailed Student’s *t*-test with STATVIEW 4.51 software (Abacus Concepts, Calabasas, CA, USA), and determined the significance of differences (*p* < 0.01).

## 3. Results

### 3.1. Osteocytes in Dmp1-Cre;Pdpn^Δ/Δ^ and Aging Mice

**Figure 1:**
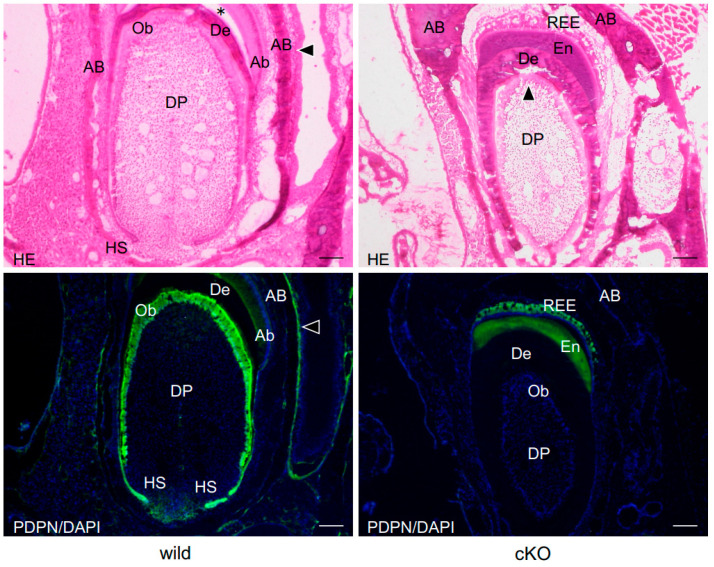
Podoplanin expression in the teeth of Dmp1-Cre;PdpnΔ/Δ mice. Sagittal sections of the lower incisors of one-week-old wild-type mice (Wild) and Dmp1-Cre;PdpnΔ/Δ mice (cKO). There were no abnormalities in the formation of alveolar bone (AB), enamel (asterisk), or dentin (De) in wild-type and cKO mice. In the immunostaining of wild-type teeth, the expression of podoplanin was detected in the odontoblast layer (Ob) at the edge of the dental pulp (DP), ameloblasts (Ab), Hertwig’s epithelial root sheath (HS), and in the periosteum (arrowhead). The expression of podoplanin was not observed in dental pulp fibroblasts (DP). In the immunostaining of podoplanin cKO teeth, the expression of podoplanin was noted in the reduced enamel epithelium (REE), but not in the dental pulp (DP) containing the odontoblast layer (Ob) or in alveolar bone (AB) containing the periosteum. There is cross-reaction to enamel (En). Bar: 100 μm.

**Figure 2:**
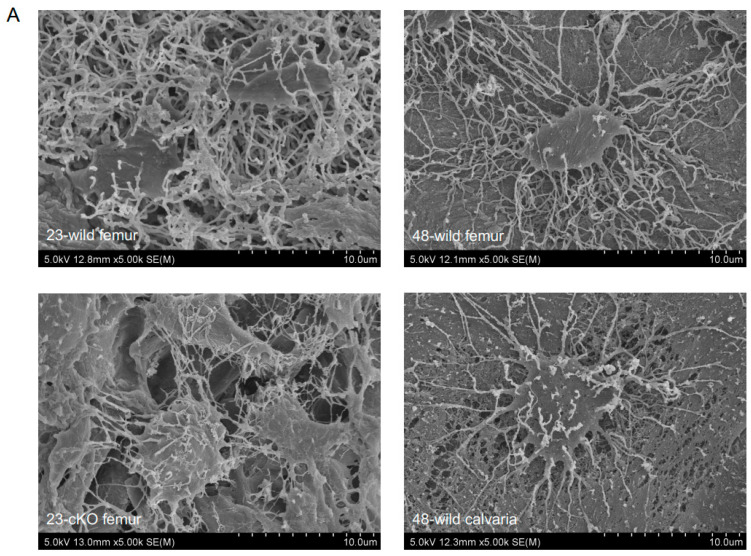

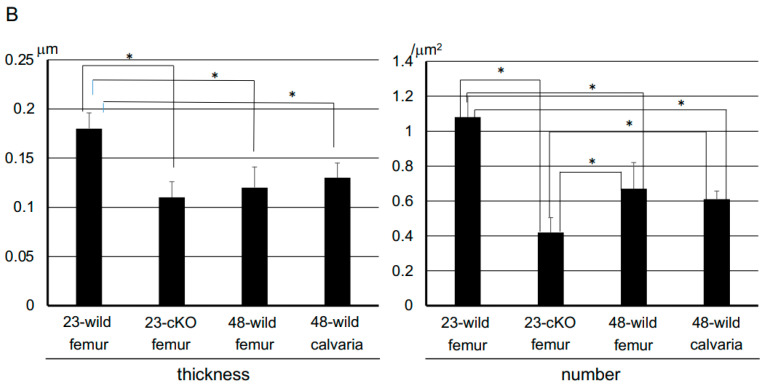
SEM analysis of osteocytes in *Dmp1-Cre;Pdpn^Δ/Δ^* and aging mice. (**A**) Microscopic observations. Osteocyte processes were well developed in the femurs of 23-week-old wild-type mice (23-wild femur). Process network formation with neighboring cells in the femur was less developed in 23-week-old *Dmp1-Cre;Pdpn^Δ/Δ^* mice (23-cKO femur) than in 23-wild femur, which was at the same developmental level as 48-week-old wild-type mouse femurs (48-wild femur) and calvarias (48-wild calvaria). (**B**) Quantitative analysis. Cell processes were significantly thinner in the femurs of 23-week-old *Dmp1-Cre;Pdpn^Δ/Δ^* mice (23-cKO femur) than in those of 23-week-old wild-type mice (23-wild femur), which was at the same developmental level as 48-week-old wild-type mouse femurs (48-wild femur) and calvarias (48-wild calvaria). The number of cell processes in the femur was significantly smaller in 23-week-old *Dmp1-Cre;Pdpn^Δ/Δ^* mice (23-cKO femur) than in 23-week-old wild-type mice (23-wild femur) and was also smaller than in 48-week-old wild-type mouse femurs (48-wild femur) and calvarias (48-wild calvaria). * Significantly different (*p* < 0.01).

The expression of podoplanin was observed in the odontoblasts and alveolar bone of 23-week-old wild-type mice, but not in 23-week-old *Dmp1-Cre;Pdpn^Δ/Δ^* mice (Figure 1). No morphological differences were observed in teeth and bones between *Dmp1-Cre;Pdpn^Δ/Δ^* mice and wild-type mice. Epithelia and nerve cells expressed podoplanin in both wild-type and *Dmp1-Cre;Pdpn^Δ/Δ^* mice (Figure 1). In SEM observations of femurs and calvarias, the osteocyte cell process network with neighboring cells was sparser in 48-week-old wild-type mice than in 23-week-old wild type mice, while it was similar in 23-week-old *Dmp1-Cre;Pdpn^Δ/Δ^* mice and 48-week-old wild-type mice (Figure 2). Intercellular spaces in the bone cells of 23-week-old *Pdpn* cKO and 48-week-old wild-type mice were markedly larger than those in 23-week-old wild-type mice, and there were fewer osteocyte cell–cell connections in bone cells from 23-week-old *Pdpn* cKO and 48-week-old wild-type mice than in those from 23-week-old wild-type mice. Furthermore, the osteocyte processes that formed were sparser and thinner and the number of networks was smaller in *Dmp1-Cre;Pdpn^Δ/Δ^* mice than in wild-type mice. In the quantitative analysis of cell process networks, there were significantly fewer osteocyte processes and thinner cell processes in femurs from 23-week-old *Dmp1-Cre;Pdpn^Δ/Δ^* mice than in those from 23-week-old wild-type mice (Figure 2). The thickness of osteocyte processes in femurs and calvarias was similar in 23-week-old *Dmp1-Cre;Pdpn^Δ/Δ^* mice and 48-week-old wild-type mice, whereas the number of cell processes in bone was significantly smaller in 23-week-old *Dmp1-Cre;Pdpn^Δ/Δ^* mice than in 48-week-old wild-type mice (Figure 2).

### 3.2. Analysis of the Absorption of Dmp1-Cre;Pdpn^Δ/Δ^ Alveolar Bone Under Mechanical Stress and Osteoclast Differentiation Markers

**Figure 3:**
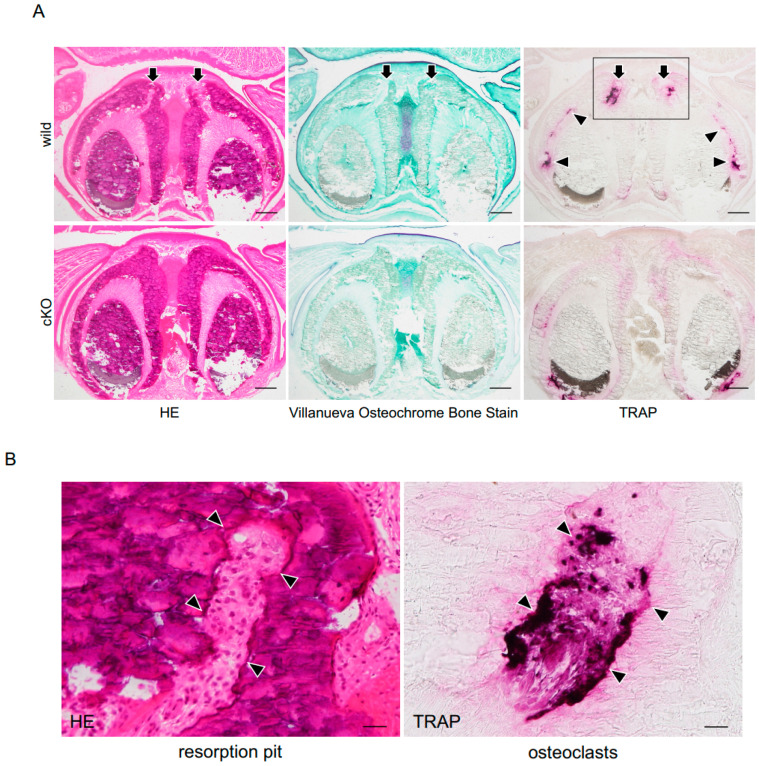
Analysis of absorption in *Dmp1-Cre;Pdpn^Δ/Δ^* alveolar bone under mechanical stress. (**A**) At a lower magnification. Morphological differences were investigated by hematoxylin and eosin (HE), Villanueva Osteochrome Bone Stain, and TRAP staining in coronal sections of 23-week-old mouse upper incisors from wild-type mice (wild) and *Dmp1-Cre;Pdpn^Δ/Δ^* mice (cKO). In the HE-stained section and adjacent section with Villanueva Osteochrome Bone Stain, no morphological abnormalities were observed in cKO. In the adjacent section of TRAP staining, the number of TRAP-positive resorption pits appeared to be higher in wild-type than in cKO mice. Cartilage formation stained in violet in alveolar bone between stressed teeth was weaker in cKO mice than in wild-type mice. Overall, no significant differences were noted in the general shape of teeth and bones; however, bone matrix formation stained in green was weak and sparse. Bar: 1 mm. (**B**) At a higher magnification of the boxes highlighted in (**A**). TRAP staining showed TRAP-positive cells in resorption pits. Bar: 100 μm.

**Figure 4:**
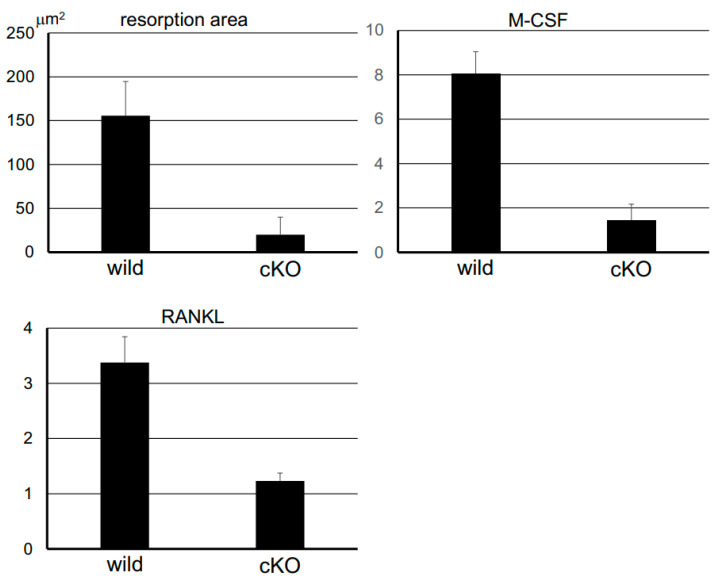
Quantitative analysis of bone resorption and osteoclast differentiation markers. The bone resorption area was calculated by Image J (National Institutes of Health, Bethesda, MD). TRAP-positive areas with bone resorption pits observed in 10-μm-thick sections were compared using the average values for the total number in 10-micron-thick 10 sections from each of the six mice. TRAP-positive resorption areas were larger in 23-week-old mouse upper incisors from wild-type mice (wild) than from *Dmp1-Cre;Pdpn^Δ/Δ^* mice (cKO). In the quantitative PCR analysis of mRNAs, the gene expression levels of RANKL and M-CSF were significantly higher in wild-type mice than in cKO mice. Gene expression levels in each sample were normalized to β-actin cDNA and expressed in arbitrary units.

In HE and Villanueva osteochrome bone staining, no morphological abnormalities were observed in *Dmp1-Cre;Pdpn^Δ/Δ^* mice (Figure 3). However, in TRAP staining, the number and spaces of TRAP-positive resorption pits in alveolar bone around teeth under mechanical stress were larger in wild-type mice than in *Dmp1-Cre;Pdpn^Δ/Δ^* mice (Figure 3). Furthermore, cartilage formation in alveolar bone between stressed teeth was markedly weaker in 23-week-old cKO mice than in 23-week-old wild-type mice. No significant differences were observed in the general shape of teeth and bones; however, bone matrix formation was weaker and sparser in cKO mice than in wild-type mice. In the quantitative analysis, the TRAP-positive resorption area in alveolar bone around the upper incisors under mechanical stress was larger in wild-type mice than in *Dmp1-Cre;Pdpn^Δ/Δ^* mice (Figure 4). In the quantitative tissue PCR analysis of mRNAs in alveolar bone tissue around teeth under mechanical stress, the gene expression levels of both RANKL and M-CSF were significantly higher in 23-week-old wild-type mice than in 23-week-old *Dmp1-Cre;Pdpn^Δ/Δ^* mice (Figure 4).

## 4. Discussion

### 4.1. Comparative Studies on Osteocyte Cell Processes Between Dmp1-Cre;Pdpn^Δ/Δ^ and Aging Mice

In the present study, we used originally established *Dmp1-Cre;Pdpn^Δ/Δ^* mice in which the floxed exon 3 of podoplanin was deleted by *Dmp1* promoter-driven Cre. Odontoblasts and osteocytes express podoplanin [1,2,3,4]. In *Dmp1-Cre;Pdpn^Δ/Δ^* mice, the expression of podoplanin was not observed in odontoblasts or alveolar bone. The present results showed that epithelia and nerve cells in *Dmp1-Cre;Pdpn^Δ/Δ^* mice expressed podoplanin (Figure 1). We previously reported the expression of podoplanin in the neural crest ectodermal mesenchyme, including odontoblasts, and in ameloblasts, meninges, nerve sheaths, osteocytes, and osteoblasts in the head and neck [4,23]. The present results indicate that the deletion of the podoplanin gene by *Dmp1* promoter region-driven Cre recombinase was successful in bone and also that the targeting of ES cells is useful. 

In SEM observations of bone, the osteocyte cell process network with neighboring cells and the thickness of cell processes were less developed in aged mice (Figure 2). Furthermore, the cell–cell connections of osteocytes were significantly sparser in podoplanin-cKO mice than in wild-type mice of the same age, and their appearance was similar to that in aged mice. These results suggest that a deficiency of podoplanin caused bone aging, and that podoplanin maintained the osteocyte network. Cell processes were clearly less developed in aged mice, suggesting that podoplanin contributes to their development in osteocytes. The intercellular spaces in osteocytes were significantly larger in podoplanin-cKO mice than in wild-type mice of the same age, and their appearance was similar to that in aged mice. Podoplanin may play a role in maintaining the bone matrix via the formation of cell processes. Podoplanin plays a key role in the extension of cell processes via the cytoplasmic portion, which induces the formation of the ezrin-radixin-moesin (ERM)-actomyosin assembly, and the binding of podoplanin to CLEC-2 induced the dissociation of the ERM-actomyosin assembly, resulting in cell shape control termed the podopanin-CELC-2 axis [21,22,23,24,25,26]. In lymph nodes, the maintenance of fibroblastic reticular cell processes is dependent on ERM phosphorylation by podoplanin because phospho-ERM induces cell process elongation via the actomyosin rearrangement in fibroblastic reticular cells [27,28]. In osteocytes, podoplanin has been suggested to contribute to the homeostasis of bone through cell process development in order to establish osteocyte communication, which contributes to the promotion of bone matrix production.

### 4.2. Comparative Studies on Bone Resorption with Mechanical Stress Between Dmp1-Cre;Pdpn^Δ/Δ^ and Wild-Type Mice 

Bone staining showed no morphological abnormalities in *Dmp1-Cre;Pdpn^Δ/Δ^* mice, indicating that podoplanin-cKO in bone is not lethal (Figure 3). However, in TRAP staining and Villanueva osteochrome bone staining, resorption in bone under mechanical stress and cartilage formation between incisors under mechanical stress were both significantly suppressed in *Dmp1-Cre;Pdpn^Δ/Δ^* mice, suggesting that the inhibition of cell process formation in osteocytes by podoplanin cKO affected the absorption and remodeling of bone under mechanical stress (Figure 3 and Figure 4). The general shapes of teeth and bone did not significantly differ; however, bone matrix formation visualized by Villanueva staining was weaker and sparser in cKO mice than in wild-type mice, suggesting that podoplanin cKO affected bone construction. In the quantitative tissue PCR analysis of mRNAs, the gene expression levels of RANKL and M-CSF were significantly higher in wild-type mice than in *Dmp1-Cre;Pdpn^Δ/Δ^* mice (Figure 4). Previous studies demonstrated that mechanical stress up-regulated the expression of M-CSF and RANKL [29,30,31]. These findings indicate that podoplanin-mediated cell process formation in osteocytes contributes to the sensing of mechanical stress and production of RANKL and M-CSF. In podoplanin-deficient bone, the deformation of osteocyte processes by mechanical stimuli may not have been recognized as stress due to the lack of cell processes as a result of podoplanin deficiency; therefore, the production of osteoclast migration/differentiation factors from activated osteocytes was not fully induced and macrophage migration to alveolar bone with mechanical stress was suppressed. The present results suggest that podoplanin-dependent osteocyte process formation indirectly plays a role in the sensing of mechanical stress to induce remodeling in bone.

## 5. Conclusions 

Podoplanin contributes to cell process formation in osteocytes to sense mechanostress and produce osteoclast migration factors. Podoplanin may play a role in the sensing of mechanical stress to induce remodeling in bone.

## Data Availability

The original contributions presented in this study are included in the article. Further inquiries can be directed to the corresponding author.
